# Impact of moderate aerobic exercise on small-world topology and characteristics of brain networks after sport-related concussion: an exploratory study

**DOI:** 10.1038/s41598-024-74474-6

**Published:** 2024-10-25

**Authors:** Jessica Coenen, Michael Strohm, Claus Reinsberger

**Affiliations:** 1 Institute of Sports Medicine, Department of Exercise and Health, Paderborn University, Warburger Straße 100, Paderborn, 33098 Germany; 2 Division of Sports Neurology and Neurosciences, Department of Neurology, Mass General Brigham, Boston, MA USA

**Keywords:** Sports injury, Concussion, Small-world topology, Graph theory, Resting-state EEG, Neurophysiology, Brain injuries

## Abstract

Sport-related concussion (SRC) is a complex brain injury. By applying graph-theoretical analysis to networks derived from neuroimaging techniques, studies have shown that despite an overall retention of small-world topology, changes in small-world properties occur after brain injury. Less is known about how exercise during athletes’ return to sport (RTS) influences these brain network properties. Therefore, in the present study dense electroencephalography (EEG) datasets were collected pre- and post-moderate aerobic exercise. Small-world properties of whole brain (WB) and the default mode network (DMN) were extracted from the EEG datasets of 21 concussed athletes and 21 healthy matched controls. More specifically, path length (LP), clustering coefficient (CP), and small-world index (SWI) in binary and weighted graphs were calculated in the alpha frequency band (7–13 Hz). Pre-exercise, SRC athletes had higher DMN-CP values compared to controls, while post-exercise SRC athletes had higher WB-LP compared to controls. Weighted WB analysis revealed a significant association between SRC and the absence of small-world topology (SWI ≤ 1) post-exercise. This explorative study provides preliminary evidence that moderate aerobic exercise during athletes’ RTS induces an altered network response. Furthermore, this altered response may be related to the clinical characteristics of the SRC athlete.

**Supplementary Information:**

The online version contains supplementary material available at 10.1038/s41598-024-74474-6.

Sport-related concussion (SRC) is a complex form of mild traumatic brain injury (mTBI) that occurs in the context of sports when biomechanical forces are directly or indirectly transmitted to the head^1^. These forces initiate a cascade of events^2^. One of which is diffuse axonal injury, particularly affecting white matter integrity and subsequently the integration of information across brain regions. Despite an expanding field of research investigating emerging neurophysiological techniques for SRC management, the results are varied^3^. In pursuit of establishing future substantiated neurophysiological approaches for SRC management, a greater understanding of how the injury and the current SRC rehabilitation strategies affect brain function is paramount.

Based on the assumption that small-world networks are globally and locally efficient^4,5^, the deterioration of small-world characteristics may elucidate deficits in integration and segregation synergies, important properties of healthy brain function^6^. In its optimal form, small-world topology can be characterized by a high clustering coefficient (CP) and short average path length (LP)^5^. The CP describes to what extent neighboring nodes connect, while the LP refers to the mean value of all possible pairs of network nodes^7^. By investigating small-world topology and network characteristics, impaired neurotransmission may be exposed by revealing altered brain function characteristics post-injury^8,9^.

Previous findings from functional magnetic resonance imaging (fMRI) and electroencephalography (EEG) studies have revealed that despite the retention of overall small-world topology, changes in small-world properties exist after brain injury^7,8,9,10,11,12^. Specifically, prior research has reported higher LP values associated with mTBI^7,9,11^, which might be an injury-induced response to a loss of functional connections^13^. Not only LP but also elevated global efficiency (which reacts inversely to LP) might be in part explained by hyperconnectivity as an adaptive compensatory mechanism to brain injuries^8,14^. In a study investigating 30 mTBI patients over a follow-up period of 3–12 months, decreased LP values from the initial visit (within 14 days post-injury) to follow-up assessment (6–12 months post-injury) were observed^11^. This may denote a reestablishment of network topology^4 ^during recovery. Conversely, in a controlled study with 22 mTBI patients, a higher CP was detected in the mTBI group, which may suggest that the brain compensates by increasing local efficiency to maintain the operations of the brain network^12,14^. Unlike the above-mentioned study, which focused on longitudinal measures, the latter focused on the acute phase post-injury by recruiting patients to the study within 12 h of injury. As local efficiency behaves proportionally to CP^6^, such an increase in CP might describe enhanced within-network communication. In contrast, a decreased CP, which has been reported during task execution after an mTBI^10^, might indicate divergence to a more random network topology for patients. A controlled study which investigated 25 mTBI patients, days within injury, reported that mTBI patients demonstrated sub-optimal network organization in the theta and alpha bands during tasks. Meanwhile, there were no group differences during resting conditions^10^.

A greater understanding of network characteristics and the prevalent network architecture post-SRC injury, has the potential to provide valuable information regarding an athlete’s post-injury brain function^8^. Building upon prior research that elicited deficits not evident in strictly resting-state conditions^15,16,17^, this study was designed to investigate the neurophysiological responses of concussed athletes to a moderate aerobic exercise test compared to a group of healthy matched controls. This exercise test selection aligns with the current return to sport (RTS) strategy, put forward by the Concussion in Sport Group^1^. Previous studies have reported favorable outcomes (e.g., expedited recovery)^18^ using aerobic exercise as a targeted intervention for post-concussion athletes^18^^,^^19^ and athletes with persistent symptoms^20^. To date, the facilitative mechanism for these favorable results remains unclear^21^. Utilizing high-density (128-channel) resting-state EEG recordings pre- and post-exercise, small-world properties for whole brain (WB) and the default mode network (DMN) were extracted via graph analysis. Through the application of graph analysis, this study aims to uncover small-world topology in the brain by quantifying the small-world index (SWI)^22^ and small-world characteristic (CP and LP) divergences between recovering concussed athletes and healthy matched controls.

## Results

### Participant characteristics

Forty-two athletes participated in this study: 21 SRC athletes (24 ± 5 years, body mass index (BMI) 24 ± 2, males *n* = 17), and 21 matched controls (24 ± 5 years, BMI 24 ± 2, males *n* = 17). Athletes participated in a range of sports, although most were involved in contact or collision sports (e.g., soccer, basketball, ice hockey and American football). There were no significant between-group differences for age, weight, height, nor BMI. The concussed athletes did have a greater number of symptoms (Mdn = 7) and greater symptom scores (Mdn = 10) than the control group (number of symptoms, Mdn = 1 and symptom score, Mdn = 1; *p* < 0.001). Despite that, all SRC athletes were medically cleared to participate in the moderate aerobic exercise test. An overview of participant characteristics is displayed in Table 1.

**Table 1:** Demographics and anthropometric characteristics. Data are presented as mean ± standard deviation, median (interquartile range), or range [in square brackets]. SRC: sports-related concussion, am. Football: American Football, ^a^: at least 365 days of no concussion or no concussion history, ^i^: independent t-test, ^m^: Mann-Whitney *U* test, ^*^: *p* < 0.05.

Characteristics	Concussed athletes	Control athletes	*p*-values (effect size)
Age (years)^m^	23.69 ± 4.98	24.03 ± 4.68	0.822 (0.070)
Height (cm)^i^	182 ± 12.37	184.48 ± 9.78	0.443 (0.239)
Weight (kg)^i^	79 ± 14.17	80.29 ± 14.31	0.821 (0.070)
Body mass index (BMI)	23.79 ± 2.10	23.38 ± 2.27	0.548 (-0.187)
Sex (male/female)	17/4	17/4	
Sport	Soccer (*n* = 6) Basketball (*n* = 6) Am. Football (*n* = 2) Other (*n* = 7)	Soccer (*n* = 7) Basketball (*n* = 6) Am. Football (*n* = 5) Other (*n* = 3)	
Number of concussions^m^	1.76 ± 1.26 [1–5]	0.62 ± 1.12 [0–4]	0.003 (-0.959)^*^
Days since last concussion	31.71 ± 41.98 [2–140]	> 365^a^	
Symptom number^m^	7 (7)	1 (2)	0.000 (-0.63)^*^
Symptom severity^m^	10 (21)	1 (3)	0.000 (-0.63)^*^

The physiological response during the exercise test was comparable between groups, with similar heart rate (HR) values at peak (moderate) exercise performance (concussed: 138 ± 8 bpm, controls: 138 ± 5 bpm, *p* = 0.859). Exercise performance was also similar between groups, with regards to maximum watts achieved (concussed: 169 ± 48 W, controls: 173 ± 48 W, *p* = 0.816), power to weight ratio (P2W, concussed: 0.50 ± 0.15 W/kg, controls: 0.47 ± 0.10 W/kg, *p* = 0.728), and duration (concussed: 13.52 ± 3.85 min, controls: 13.81 ± 3.85 min, *p* = 0.816). For an overview of exercise performance see Table 2. All participants achieved their individual moderate intensity threshold (70% of their maximum HR) without prematurely terminating the exercise test due to symptom exacerbation or voluntary choice.

**Table 2:** Exercise performance parameters: pre-, at moderate exercise intensity, and post- exercise. Data are presented as mean ± standard. HR: heart rate, bpm: beats per minute, P2W: power to weight ratio, W/kg: watts per kilogram, min: minutes, Student t-tests, ^*^: *p <* 0.05.

	Parameter	Concussed athletes	Controls athletes	*p*-value (effect size)
**pre-Exercise**	HR (bpm)	71.05 ± 10.40	74.86 ± 12.63	0.304 (0.321)
**moderate -**	HR (bpm)	137.86 ± 7.83	137.48 ± 5.41	0.859 (-0.055)
**Exercise**	Watts (W)	169.05 ± 48.12	172.62 ± 48.12	0.816 (0.072)
	P2W (W/kg)	0.50 ± 0.15	0.47 ± 0.10	0.728 (-0.108)
	Time (min)	13.52 ± 3.85	13.81 ± 3.85	0.816 (0.072)
**post-Exercise**	HR (bpm)	106.86 ± 11.37	106.95 ± 9.88	0.978 (0.009)

### Small-world characteristics

Mixed analysis of variance (ANOVA) were performed on the binary and weighted graph parameters (LP, CP, SWI), for each of the networks (WB, DMN) to explore interactions between group and exercise. For WB-LP there was a significant interaction (F_(1,40)_ = 5.258, η² = 0.116, *p* = 0.027). Further between group analysis revealed that post-exercise, concussed athletes (2.14 ± 0.07) had significantly higher WB-LP than the control group (2.10 ± 0.06) (*t*(40)= -2.108, *p* = 0.041, *d* = -0.65), reflecting a medium effect. For WB-SWI, there was a significant main effect for exercise (F_(1,40)_ = 6.147, η² = 0.133, *p* = 0.023), in addition to a significant interaction between exercise and group (F_(1,40)_ = 5.618, η² = 0.123). Exploring the impact of exercise within each group revealed that control athletes’ WB-SWI significantly increased from pre- (51.14 ± 4.73) to post-exercise (53.25 ± 3.61; Z = -2.833, *p* = 0.003, *r* = − 0.05), while concussed athletes’ index also increased, however, it did not do so significantly (*p* = 0.875). There was also a significant interaction between exercise and group, for DMN-CP at a threshold of 10% in the binary analysis (F(1,40) = 4.551, η² = 0.132) and weighted analysis (F(1,40) = 5.720, η² = 0.160). Further between group analysis revealed that binary pre-exercise DMN-CP values were significantly higher in the concussed group (0.25 ± 0.11, 0.06) than controls (0.17 ± 0.09; U = 91, Z = -2.645, *p* = 0.007, *r* = − 0.44), with a medium effect size. For the weighted analysis, results were comparable with the concussed group (0.20 ± 0.12) showing a significantly higher CP than matched controls (0.12 ± 0.06; t(40) = -2.476, *p* = 0.018, d = − 0.81), with a large effect size. An interaction between exercise and group also showed for DMN-LP (F(1,40) = 5.150, η² = 0.114). However, there were no significant between group differences pre- (*p* = 0.709) neither post-exercise (*p* = 0.249). Table 3 presents an overview of the significant interaction results, between exercise and group, while more detailed tables with all the ANOVA test results are provided in the supplementary material.

**Table 3:** Overview of significant interactions identified by the mixed-ANOVA analyses. WB: whole brain, DMN: default mode network, B: binary graph, W: weighted graph, η²: Partial Eta squared.

Network Parameter	Graph - threshold	Concussed athletes	Control athletes	mixed-ANOVA	*p*-value
WB-LP	B − 20	_pre_ 2.13 ± 0.08 _post_ 2.14 ± 0.07	_pre_ 2.13 ± 0.10 _post_ 2.10 ± 0.06	F_(1,40)_ = 5.258, η² = 0.116	0.027
WB-SWI	W − 50	_pre_ 51.03 ± 4.06 _post_ 51.08 ± 4.69	_pre_ 51.14 ± 4.73 _post_ 53.25 ± 3.61	F_(1,40)_ = 5.618, η² = 0.123	0.023
DMN–CP	B -10	_pre_ 0.248 ± 0.11 _post_ 0.202 ± 0.07	_pre_ 0.170 ± 0.09 _post_ 0.198 ± 0.09	F_(1,40)_ = 4.551, η² = 0.132	0.041
DMN–CP	W − 10	_pre_ 0.199 ± 0.12 _post_ 0.167 ± 0.07	_pre_ 0.122 ± 0.06 _post_ 0.153 ± 0.08	F_(1,40)_ = 5.720, η² = 0.160	0.023
DMN-LP	W − 40	_pre_ 3.52 ± 0.60 _post_ 3.32 ± 0.54	_pre_ 3.46 ± 0.48 _post_ 3.48 ± 0.38	F_(1,40)_ = 5.150, η² = 0.114	0.029

In examining the association between the presence of small-world topology and SRC, the results of the chi-square tests of independence showed a significant association between SRC and the absence of small-world topology (SWI ≤ 1) in the weighted WB post-exercise with a threshold of 40% (𝝌(1,42) = 5.676, *p* = 0.048, 𝜑 = -0.368).

## Discussion

The present study aimed to investigate small-world topology and network characteristics in a group of SRC athletes during their recovery process compared to healthy matched control athletes to explore functional brain network characteristics in resting-state and in response to moderate aerobic exercise. Pre-exercise SRC athletes had higher DMN-CP values compared to controls, while post-exercise SRC athletes had higher WB-LP compared to controls. Post-exercise, there was also a significant association identified between the SRC group and an absence of small-world topology.

Higher LP in WB for the SRC group after the exercise stimulus may be the result of decreased global connectedness, which corresponds to the findings of Cao et al.^7^ and Li et al.^9^, who also reported an enhanced LP (along with an increased CP) as a result of mTBI. Comparable results have also been described in the context of neurological disorders due to structural alterations^23^. A clear deterioration from a small-world-like network configuration has been described for patients with neurological disorders^23^. Suggesting that these results reflect injury-induced pathophysiology. On the other hand, it may reflect an adaptive compensatory neuronal mechanism. Neural networks may require an increased connectivity response post-injury to maintain necessary communication throughout the network. But whether and how this adaptive mechanism, proposed by Hilary et al.^14^ as hyperconnectivity, contributes to the results of the present study remains unclear. As no between-group differences were revealed in the resting-state condition pre-exercise, it is possible that the stress of moderate aerobic exercise elicited a post-injury compensatory mechanism, which is otherwise not necessary during rest. However, it is difficult to confirm this interpretation due to a lack of previous research, which investigated athletes’ neural network response to exercise during their RTS process.

The significant interaction between exercise and group for WB-LP may be better interpreted in relation to the findings in healthy athletes^24,25,26^. In a study by Tamburro et al.^24^, functional network properties in the alpha frequency band responded to an exhaustive cycling task by first increasing global efficiency, which may reflect an increase in alertness and preparedness, followed by a decrease in efficiency as the participants achieved exhaustion. Furthermore, Büchel et al.^26^ reported similar results such that low to moderate intensity exercise appears to enhance network characteristics, while exhaustive exercise levels impair network properties. In the present study, in response to moderate aerobic exercise, the healthy control athletes displayed lower LP values than the SRC group in the WB. This may resemble better connectedness, matching the result of enhanced global efficiency reported by Tamburro et al.^24^. In contrast, the SRC group showed higher LP values, which is possibly related to decreased connectivity as a sign of functional disconnection^13^. Nevertheless, whether an increased LP that is induced by moderate aerobic exercise is indeed a direct result of functional brain injury needs to be further explored in larger longitudinal studies.

A significant association between SRC and the absence of small-world topology (SWI ≤ 1) was revealed post-exercise in the weighted WB analysis at a threshold of 40%. The absence of small-world topology may imply that SRC athletes exhibit less efficient informational exchange through less segregated information processing, less integrated information processing, or both^6,13^. In exploring this result further, the absence of small-world topology (SWI ≤ 1) post-exercise resulted for five SRC athletes. These athletes were either in the early acute recovery phase post-injury with a positive concussion history or had a cumulative concussion history with a high symptom severity score. The majority of SRC athletes recruited for this study were already in the subacute phase post-injury (i.e., 8–89 days)^27^, but three athletes (/5 SRC athletes with SWI ≤ 1) were in the earlier acute phase of RTS (i.e., 2–7 days post-injury). The other two SRC athletes (/5 SRC athletes with SWI ≤ 1) were characterized by a greater number of previous concussions or a combination of previous concussion history with an increased symptom severity score. An overview of participant characteristics for these five SRC athletes can be found in Table 4. Although the low number of subjects did not allow further statistical investigations, these observations may imply that an athlete’s concussion history (i.e., days since injury as well as number of previous concussions) and symptom severity scores have an impact on (the absence of) small-word topology, or the absence thereof. However, the persistence of this connection throughout an athlete’s recovery may diminish over time, and therefore should be explored with longitudinal studies from the early acute phase (i.e., 2–7 days post-injury) or especially in complex cumulative concussion cases^28^.

**Table 4:** Demographics and participant characteristics of the SRC athletes without small-world topology. Five concussed athletes post-exercise had a less than 1 value for SWI (absence of small world topology) in the weighted WB at a threshold of 40%.

Characteristics	Athlete 1	Athlete 2	Athlete 3	Athlete 4	Athlete 5
Age (years)^m^	29.61	18.75	18.5	30.98	27.53
Height (cm)^i^	183	178	183	212	177
Weight (kg)^i^	81	70	74	109	79
Sex (male/female)	Male	Male	Male	Male	Male
Sport	Basketball	Soccer	Soccer	Basketball	American Football
Number of concussions	1	1	1	5	4
Days since last concussion	4	2	7	19	116
Symptom number	1	1	7	12	17
Symptom severity	1	4	14	28	52

Meanwhile, for the DMN, there was an interaction effect observed for both CP and LP, which suggests exercise plays a role in modulating the network dynamics in this subnetwork. However, the post-hoc tests reported significantly higher CP values (binary and weighted) for the SRC group compared to the matched controls. For the DMN-LP, only within the concussed group was there a significant reduction from pre- to post-exercise. Within the control group the DMN-LP values slightly increased in response to exercise. Higher CP values post-injury have already been described and discussed in previous reviews on TBI^29,30,31^. Enhanced local interconnectedness may represent a compensatory process that is used to re-establish regional network communication or to ensure fault-tolerance on local levels^6,14^. There is also the idea that it may reflect a failure to deactivate the DMN during goal-directed behaviour^30^. Still, the clinical relevance of greater connectedness within the DMN remains unclear^32^ and warrants further investigation.

The impact of the moderate aerobic exercise stimulus in this study on the DMN-LP of the SRC-athletes, may imply an enhancement of information transfer within this network^33^. Evidence from a longitudinal study suggests a decreased LP during RTS may indeed reflect recovery. Churchill et al.^8^ explored small-world organization of brain networks in 26 concussed athletes across three time points post-injury. First measurements were taken acutely (within 1 week of concussion), then again once the athlete has completed their RTS process, and as follow-up, one year after their RTS. Results revealed elevated global efficiency acutely post-injury, which then significantly resolved by the time the concussed athletes were medically cleared (i.e., determined by symptom resolution) to RTS. Relating this back to the present study, the moderate aerobic exercise stimulus appears to induce a recovery facilitative mechanism. These result may in part contribute to a greater understanding of why moderate aerobic exercise interventions post-SRC lead to favorable results.

This cohort study is not without limitations. First, the sample was rather small and heterogeneous. Replication of these results in a larger sample would greatly strengthen these conclusions. A second limitation is that the datasets were collected at one point in time, along a potential course of recovery. A future longitudinal study may consider using graph theoretical analysis to explore recovering SRC athletes’ response to moderate aerobic exercise before and after a guided RTS intervention to further reveal facilitative mechanism for the favorable results of exercise post-injury. Methodological limitations also need to be considered such that the DMN only resembles a relatively small graph. The brain connectivity toolbox (BCT) may only have a limited capacity to calculate some graph parameters in small networks, which may have led to the zero-values for CP (and therefore SWI) in the DMN for a few athletes. This may also present a potential bias towards a shorter LP^5,34^. However, possible capacity limitations of BCT would have affected both groups. Second, by only comparing brain networks to surrogate random graphs, this study did not account for the affinity to lattice networks (depicted by a high CP and LP), an organizational topology approximated in TBI^27^. Therefore, other graph theoretical measures that differentiate between small-world and lattice networks would further supplement the interpretation of these results^22^. Despite this possible limitation of SWI, the choice for the SWI and use of BCT was purposefully considered so that reproducibility of results and comparisons between studies is amicable^35^. Similar consideration was enforced in choosing a conventional applied statistical approach, which may encourage further investigations.

Quantitative EEG applications may particularly provide important insights into the pathology of SRC (e.g., the neurometabolic, neurophysiologic, and microstructural alterations post-injury)^36,37^. However, the lack of consistency and replicability of any one technique still limits consensus and a broader clinical use^36,37^. This study may provide a basis by which future investigations can replicate and expand on the utilization of graph theoretical analysis throughout the recovery process after SRC to reflect not only pathology but also mechanisms for recovery.

In summary, this explorative study provides preliminary evidence that moderate aerobic exercise during athletes’ recovery post-SRC induces an altered network response. This includes higher LP values post-exercise in the WB network for SRC athletes compared to healthy matched controls. In addition to a statistically significant association between SRC and the absence of small-world topology in the WB. Small-world topology might be particularly sensitive to the impact of moderate aerobic exercise during the acute recovery phase or to the effects of cumulative concussion history combined with high symptom severity. Graph theoretical analysis may therefore elucidate compensatory, while also adaptive responses to moderate aerobic exercise during an athlete’s RTS process. Although the clinical relevance of these findings still needs to be interpreted with caution, they may encourage further investigations into small-world topology with a larger sample and a longitudinal design.

## Methods

### Participants

Twenty-one SRC athletes were recruited for this exploratory cohort study from the Sports Medicine Institute at Paderborn University, Germany. For comparison 21 healthy matched (via. sex, age, height, weight, and sport-type) control athletes were also recruited. The athletes in the SRC group had to have been clinically diagnosed with a sports-related concussion based on the concussion in sport guidelines^1^. Following diagnosis, in order to partake in the exercise test, SRC athletes must have received medical clearance to initiate their RTS strategy. In accordance with the current RTS strategy the SRC athletes recruited for this study were between stage 2 (i.e., mild to moderate aerobic exercise) and stage 5 (i.e., sport specific training)^1,19^. In order to get a sample of athletes who were within their RTS strategy, those who had completed it (i.e., returned to unrestricted sports participation) were excluded from this study. Inclusion criteria for the control athletes were as follows: they must not have experienced a concussion or mTBI within the past year (i.e., > 365 days since last concussion) and must be active athletes in their sport (i.e., regularly participating). The study was conducted in accordance with the Declaration of Helsinki and approved by the ethics committee of the Westphalian Medical Board (2018-522-f-S). Written informed consent was obtained from each participant. The trial was registered at the German Clinical Trial Register (DRKS00029207).

### Experimental procedure

**Figure 1:**
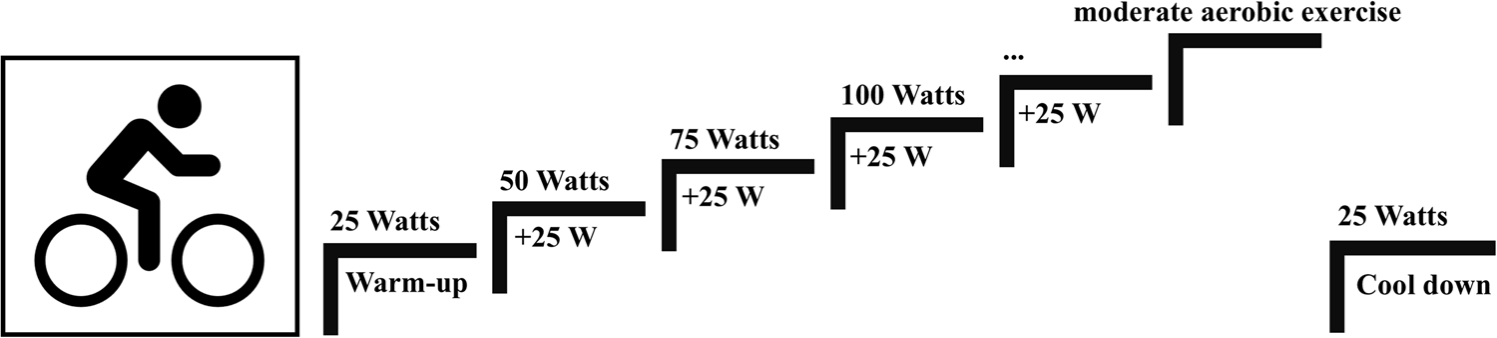
Standardized moderate aerobic bike exercise test.

Before the first resting-state EEG, participants were asked to fill out a questionnaire collecting information regarding sports background, concussion history, and current symptom severity. Symptom severity was obtained via the symptom checklist of the SCAT5 (Standardized Concussion Assessment Tool, 5th Edition)^38^. Biometric measurements of height, weight, and BMI were also collected from the participants. The EEG measurements were taken in a dimly lit room, with the participants in the supine position and their eyes closed. A 128-channel EEG (actiCHamp, Brain Products GmbH, Gilching, Germany) was set on the head of the participants according to the 10–10 system. Electrode locations on the scalp were recorded using Captrak (Brain Products GmbH) scanner and software. Impedances were constantly kept below 25kΩ. FPz served as the ground electrode, while FCz served as the reference electrode. The data were recorded at a sampling rate of 1000 Hz. Between the two (pre- and post-) resting-state EEG measurements, participants were exposed to a standardized moderate aerobic bike exercise test (Fig. 1). The exercise bike test started with a 2-minute warm-up at 25 watts and a pace between 30 and 60 rpm (revolutions per minute). Every 2 min the power output increased by 25 watts. The pace increased after 4 min to between 60 and 70 rpms and at 8 min to between 70 and 90 rpms, then remained consistent. The bike exercise test progressed until the athletes reached 70% of their age-dependent maximum HR^15,16^. To obtain the athletes’ individual moderate intensity threshold, 70% of the maximum HR determined by the equation: 0.7*(208 – (0.7*age))^39^was calculated. HR was monitored with a 12-lead electrocardiogram (Custo cardio 100 BT, Custo Med) throughout the exercise test. As a precautionary measure, a visual analogue scale (VAS) for symptoms (0 = feel terrific, no symptoms, to 10 = feel terrible) was applied every 2 min throughout the exercise test to monitor eventual symptom elevation. In line with the RTS strategy^1^,^19^ and previous studies^18^,^40^, if more than mild exacerbation (i.e., more than 3 points) of symptoms occurred, the test was to be terminated. Once the exercise goal of moderate intensity (70% the athletes individual maximum HR) was achieved, power output was brought back down to 25 watts and the athletes were instructed to slow their pace to 50 rpms for a cool-down, which lasted 2 min.

### Data processing and graph analysis

Using BrainVision Analyzer (BrainVision Analyzer, Version 2.1.2, Brain Products GmbH, Gilching, Germany) EEG datasets were downsampled to 250 Hz^41^. The datasets were checked for electrode bridges by drawing on the MATLAB-based (MATLAB, Version 2019b, Mathworks, Natick, Massachusetts) eBridge script^42^ and the magnitude-squared coherence in BrainVision Analyzer. For this, coherences above 0.9 were defined as electrode bridges. If both the eBridge function as well as the magnitude-squared coherence detected an electrode bridge, the channels involved were interpolated by Topographic Interpolation by Spherical Splines. Subsequently, a zero-phase shift Butterworth Filter with a low cutoff of 1 Hz (time constant (s): 0.1591549, Order 4) and a high cutoff of 30 Hz (Order 4) was applied. A notch filter (50 Hz) to handle the local grid line noise was also applied. Still noisy channels were adjusted by Topographic Interpolation by Spherical Splines. The dataset was then re-referenced to a common average reference. Eye and electrocardio artifacts were corrected using Independent Component Analysis based on BrainVision Analyzer software. The remaining artifacts were marked manually and the first four artifact-free segments were exported for further analysis^43^.

Using Brainstorm software^44^, the functional connectivity parameter phase locking value (PLV)^45^was computed. Colin27 template was used as the default anatomy and the individual head shape was approximated based on electrode positions captured with CapTrak. An identity matrix was used as noise covariance. Drawing on the Boundary Element Method, the head model was calculated^46^. The sources were reconstructed using minimum-norm imaging. A functional connectivity matrix was generated for each of the four segments and then one averaged matrix was exported for the alpha frequency band (7–13 Hz) for each participant^47^. The deliberate decision to conduct the analysis exclusively in the alpha frequency was made as previous research has demonstrated its utility to reflect disconnection in neurological diseases^13,48^. Further, PLV-based network metrics are strongly associated with alpha relative power, clustering coefficient and betweenness centrality^49^.

**Figure 2:**
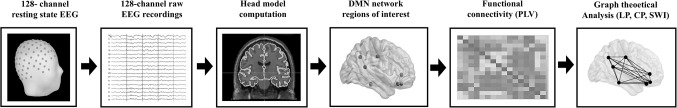
Data processing pipeline for the DMN network. Before the resting-state EEG measurement, the electrode locations on the scalp were obtained using Captrak. After the resting-state EEG measurement, the collected dataset was pre-processed in BrainAnalyzer software. In preparation for functional connectivity analysis, a head model was computed in Brainstorm, run through MATLAB. Next, the ROIs corresponding to the DMN network were exclusively selected (according to Kabbara et al.)^47^. PLV between the regions of the network was calculated as a measure of functional connectivity. The resulting matrix was exported and graph (/small world) characteristics such as SWI were computed using BCT^4^. EEG: electroencephalography, ROIs: regions of interest, DMN: default mode network, PLV: Phase Locking Value, SWI: small-world index, BCT: Brain Connectivity Toolbox. Some of the above images were generated with the help of BrainNet Viewer^53^.

Using the Desikan-Killiany atlas^50^ the ROIs for each of the networks were selected. The WB analysis took all 68 areas of the atlas into account, while 14 ROIs were selected for the DMN based on Kabbara et al.^47^. Regions of the medial prefrontal cortex, medial temporal lobe, posterior cingulate cortex, and parietal cortex were defined and used for the DMN analyses. This network was selected a priori because the DMN is known as a task-negative network, characterized by increased activity during resting-state conditions^51^, and because previous research has shown that this network may be particularly impacted by physical stress after a SRC injury^15^. For a list of the ROIs included within the Desikan-Killiany atlas please refer to the supplementary material. The PLV-based adjacency matrices were thresholded proportionally from 10 to 50% in increments of 10% to include several gradations of possibly-influential connections, in-line with previously published graph analysis^33,34^. The matrices were analyzed binarized and weighted, increasing the contrast and containing more information, respectively^34^. Thus, elucidating specific network properties while testing the stability of the networks^52^. The graph-theoretical parameters for the analysis, CP and LP, were computed using the MATLAB-based Brain Connectivity Toolbox (BCT)^5^. The normalized CP ($\:\gamma\:$) and LP ($\:\lambda\:$), required for the SWI-calculation, were determined with respect to random surrogates derived from the original networks^5,22^. Small-world topology was evaluated based on the following formula: SWI $\:\left(\sigma\:\right)=\frac{\gamma\:}{\lambda\:}$^22^. Networks with values higher than 1 depict small-world topology^22^. The key data processing steps are again portrayed in Fig. 2. After conducting the initial graph analysis, 4 datasets pre-exercise and 6 datasets post-exercise for the DMN had to be excluded from further analysis. This was due to zero-values resulting from the calculation of CP and, consequently, for SWI.

### Statistical analysis

The statistical analyses were performed using IBM SPSS Statistics (Version 29, Armonk, NY: IBM Corp). The data were checked for normality according to the Shapiro-Wilk test. Independent *t*-tests were computed to determine between-group mean differences for participant characteristics (age, height, weight) and exercise performance (HR, watts, P2W and duration), if data was normally distributed, otherwise, the Mann-Whitney *U* test was used as the non-parametric alternative. Levene’s test of homogeneity of variances was used to ensure the correct interpretation of the independent *t*-test statistics. To investigate brain function divergences between groups, in response to exercise, a mixed-ANOVA was performed on the binary and weighted graph parameters. Additionally, chi-square tests of independence were conducted to examine the association between the presence of small-world topology and SRC. If the expected cell frequency in the chi-square test was lower than five, the Monte Carlo method with 10,000 samples was used to estimate the significance. Given the exploratory nature of this study, the significance level was set to 𝛼 = 0.05. There were no further adjustments made for the multiple statistical tests conducted across different thresholds. Despite the increased risk of incorrectly identifying significant effects, this approach prioritizes the identification of potential associations for further investigations^54^. The effect size Cohen’s *d* was computed for normally distributed data, Pearson’s *r* for non-normally distributed or non-metric data, and the phi-coefficient for the results of the chi-square test^52^.

## Supplementary Information

Supplementary Material 1.

## Data Availability

The datasets collected and analyzed during this study are available from the corresponding author upon reasonable request. In addition, the standard operating procedure (SOP) for the exercise test, as well as the SOP for the resting-state measurements are available upon request.
