# Supplementary material for: Impact of moderate aerobic exercise on small-world topology and characteristics of brain networks after sport-related concussion: an exploratory study
Source: Sci Rep. 2024 Oct 25;14:25296. doi: 10.1038/s41598-024-74474-6 (PMC11511817; doi:10.1038/s41598-024-74474-6)
Supplement: Supplementary file 1 — Supplementary Material 1. [file 41598_2024_74474_MOESM1_ESM.docx]

**Impact of moderate aerobic exercise on small-world characteristics of brain networks after sport-related concussion: an exploratory study**

*Jessica Coenen ^1Ɨ^; Michael Strohm ^1Ɨ^; Claus Reinsberger ^1,2*^*

# Supplementary Information

| WB  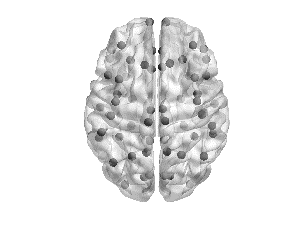  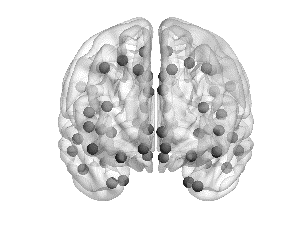  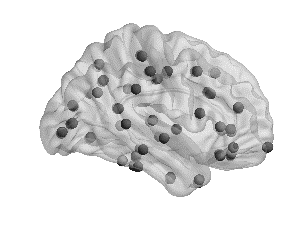 | bankssts (BAN)^L/R^, caudal anterior cingulate (CAC)^L/R^, caudal middle frontal (CMF)^L/R^, cuneus (CUN)^L/R^, entorhinal (ENT)^L/R^, frontal pole (FRP)^L/R^, fusiform (FUF)^L/R^, inferior parietal (INP)^L/R^, inferior temporal (INT)^L/R^, insula (INS)^L/R^, isthmus cingulate (ISC)^L/R^, lateral occipital (LAO)^L/R^, lateral orbitofrontal (LOF)^L/R^, lingual (LIN)^L/R^, medial orbitofrontal (MOF)^L/R^, middle temporal (MIT)^L/R^, paracentral (PAC)^L/R^, parahippocampal (PHC)^L/R^, pars opercularis (PAR)^L/R^, pars orbitalis (POR)^L/R^, pars triangularis (PST)^L/R^, pericalcarine (PER)^L/R^, postcentral (POC)^L/R^, posterior cingulate (PCC)^L/R^, precentral (PEC)^L/R^, precuneus (PCU)^L/R^, rostral anterior cingulate (RLC)^L/R^, rostral middle frontal (RMF)^L/R^, superior frontal (SUF)^L/R^, superior parietal (SUP)^L/R^, superior temporal (SUT)^L/R^, supramarginal (SUM)^L/R^, temporal pole (TEP)^L/R^, transverse temporal (TTE)^L/R^ |
| --- | --- |
| DMN  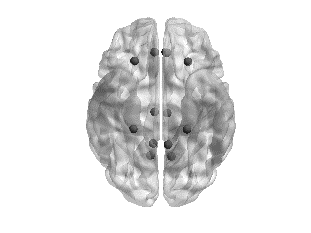  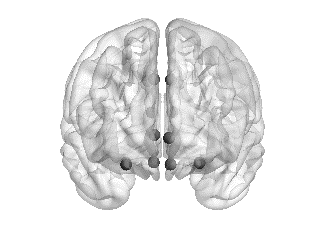  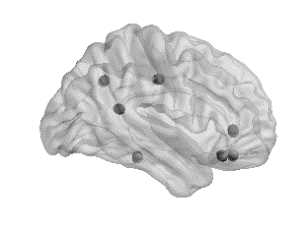 | isthmus cingulate (ISC)^L/R^, medial orbitofrontal (MOF)^L/R^, posteriorcingulate (PCC)^L/R^, precuneus (PCU)^L/R^, rostral anterior cingulate (RLC)^L/R^, lateral orbitofrontal (LOF)^L/R^, parahippocampal (PHC)^L/R^ |

**Supplementary Table 1.** Regions of interest (ROI) from Desikan Killany atlas^1^ used for the functional connectivity matrix in Brainstorm software. WB: whole brain, DMN: default mode network. Image created in BrainNet Viewer.^2^

|  | **Parameter** | **Concussed athletes** | **Controls athletes** | **p-value** | **Cohen’s d^a^/  r (effect size)^b^** |
| --- | --- | --- | --- | --- | --- |
| **Pre-Exercise** | HR (bpm) | 71.05 ± 10.40 | 74.86 ± 12.63 | 0.304^a^ | 0.321^a^ |
|  | RPE | 7.43 ± 2.46 | 7.05 ± 1.21 | 0.538^b^ | -0.04^b^ |
|  | Symptoms (VAS) | 0.86 ± 0.94 | 0 ± 0 | 0.000^b**^ | -0.65^b^ |
| **Moderate** | HR (bpm) | 137.86 ± 7.83 | 137.48 ± 5.41 | 0.859^a^ | -0.055^a^ |
| **Exercice** | RPE | 12.62 ± 2.52 | 13.19 ± 2.28 | 0.453^b^ | -0.12^b^ |
|  | Symptoms (VAS) | 1.81 ± 1.84 | 0 ± 0 | 0.000^b**^ | -0.65^b^ |
|  | Watts (W) | 169.05 ± 48.12 | 172.62 ± 48.12 | 0.816^a^ | 0.072^a^ |
|  | P2W (W/kg) | 0.50 ± 0.15 | 0.47 ± 0.10 | 0.728^a^ | -0.108^a^ |
|  | Time (min) | 13.52 ± 3.85 | 13.81 ± 3.85 | 0.816^a^ | 0.072^a^ |
| **Pre-Exercise** | HR (bpm) | 106.86 ± 11.37 | 106.95 ± 9.88 | 0.978^a^ | 0.009^a^ |
|  | RPE | 9.90 ± 2.21 | 8.71 ± 1.64 | 0.062^b^ | -0.29^b^ |
|  | Symptoms (VAS) | 1.42 ± 1.43 | 0 ± 0 | 0.000^b**^ | -0.67^b^ |

**Supplementary Table 2.** Exercise performance parameters - pre-, during and post- moderate exercise. HR: heart rate, bpm: beats per minute, RPE: rate of perceived exertion, VAS: visual analog scale, P2W: power to weight ratio, min: minutes, ^a^ Student t-test, ^b^ Mann- Whitney test, mean ± standard deviation, ^*^ p < 0.05, ^**^ p < 0.001

| **Network** | **Parameter** | **Graph - threshold** | **Test statistic** | **P-value** |
| --- | --- | --- | --- | --- |
| **WB** | CP | B – 10 | F_(1,40)_ = 3.368, η² = .078 | 0.074 |
| **WB** | CP | B – 20 | F_(1,40)_ = 0.060, η² = .002 | 0.807 |
| **WB** | CP | B – 30 | F _(1,40)_ = 0.835, η² = .200 | 0.366 |
| **WB** | CP | B – 40 | F _(1,40)_ = 0.012, η² = .000 | 0.915 |
| **WB** | CP | B – 50 | F _(1,40)_ = 0.187, η² = .005 | 0.668 |
| **WB** | LP | B – 10 | F _(1,40)_ = 0.044, η² = .001 | 0.835 |
| **WB** | LP | B – 20 | F _(1,40)_ = 5.258, η² = .116 | 0.027* |
| **WB** | LP | B – 30 | F _(1,40)_ = 0.226, η² = .006 | 0.637 |
| **WB** | LP | B – 40 | F _(1,40)_ = 0.120, η² = .003 | 0.761 |
| **WB** | LP | B – 50 | F _(1,40)_ = 0.018, η² = .004 | 0.674 |
| **WB** | SWI | B – 10 | F _(1,40)_ = 1.462, η² = .035 | 0.234 |
| **WB** | SWI | B – 20 | F _(1,40)_ = 1.367, η² = .033 | 0.249 |
| **WB** | SWI | B – 30 | F _(1,40)_ = 1.156, η² = .028 | 0.289 |
| **WB** | SWI | B – 40 | F _(1,40)_ = 2.515, η² = .059 | 0.121 |
| **WB** | SWI | B – 50 | F _(1,40)_ = 1.181, η² = .029 | 0.284 |
| **DMN** | CP | B – 10 | F _(1,40)_ = 4,551, η² = .132 | 0.041* |
| **DMN** | CP | B – 20 | F _(1,40)_ = 0.019, η² = .000 | 0.892 |
| **DMN** | CP | B – 30 | F _(1,40)_ = 2.743, η² = .064 | 0.106 |
| **DMN** | CP | B – 40 | F _(1,40)_ = 0.232, η² = .006 | 0.631 |
| **DMN** | CP | B – 50 | F _(1,40)_ = 0.686, η² = .017 | 0.411 |
| **DMN** | LP | B – 10 | F _(1,40)_ = 0.000, η² = .000 | 0.988 |
| **DMN** | LP | B – 20 | F _(1,40)_ = 0.398, η² = .001 | 0.535 |
| **DMN** | LP | B – 30 | F _(1,40)_ = 0.078, η² = .002 | 0.782 |
| **DMN** | LP | B – 40 | F _(1,40)_ = 2.526, η² = .059 | 0.120 |
| **DMN** | LP | B – 50 | F _(1,40)_ = 1.588, η² = .038 | 0.215 |
| **DMN** | SWI | B – 10 | F _(1,40)_ = 3.214, η² = .097 | 0.083 |
| **DMN** | SWI | B – 20 | F _(1,40)_ = 0.025, η² = .001 | 0.874 |
| **DMN** | SWI | B – 30 | F _(1,40)_ = 1.037, η² = .025 | 0.315 |
| **DMN** | SWI | B – 40 | F _(1,40)_ = 0.078, η² = .002 | 0.782 |
| **DMN** | SWI | B – 50 | F _(1,40)_ = 0.562, η² = .458 | 0.458 |

**Supplementary Table 3**. Overview of the mixed ANOVA test statistics for the binary analysis.
WB: whole brain, DMN: default mode network, B: binary graph, W: weighted graph, ^*^: p < .05.

| **Network** | **Parameter** | **Graph - threshold** | **Test statistic** | **P-value** |
| --- | --- | --- | --- | --- |
| **WB** | CP | W – 10 | F _(1,40)_ = 1.761, η² = .042 | 0.192 |
| **WB** | CP | W – 20 | F _(1,40)_ = 0.092, η² = .002 | 0.764 |
| **WB** | CP | W – 30 | F _(1,40)_ = 0.877, η² = .021 | 0.355 |
| **WB** | CP | W – 40 | F _(1,40)_ = 0.117, η² = .003 | 0.734 |
| **WB** | CP | W – 50 | F _(1,40)_ = 0.025, η² = .001 | 0.874 |
| **WB** | LP | W – 10 | F _(1,40)_ = 0.001, η² = .000 | 0.981 |
| **WB** | LP | W – 20 | F _(1,40)_ = 1.744, η² = .042 | 0.194 |
| **WB** | LP | W – 30 | F _(1,40)_ = 0.452, η² = .011 | 0.505 |
| **WB** | LP | W – 40 | F _(1,40)_ = 0.370, η² = .009 | 0.546 |
| **WB** | LP | W – 50 | F _(1,40)_ = 0.424, η² = .010 | 0.519 |
| **WB** | SWI | W – 10 | F _(1,40)_ = 1.322, η² = .032 | 0.257 |
| **WB** | SWI | W – 20 | F _(1,40)_ = 1.908, η² = .046 | 0.175 |
| **WB** | SWI | W – 30 | F _(1,40)_ = 1.933,η² = .046 | 0.172 |
| **WB** | SWI | W – 40 | F _(1,40)_ = 3.983, η² = .091 | 0.053 |
| **WB** | SWI | W – 50 | F _(1,40)_ = 5.518, η² = .123 | 0.023* |
| **DMN** | CP | W – 10 | F _(1,40)_ = 5.720, η² = .160 | 0.023* |
| **DMN** | CP | W – 20 | F _(1,40)_ = 0.087, η² = .002 | 0.770 |
| **DMN** | CP | W – 30 | F _(1,40)_ = 3.744, η² = .086 | 0.060 |
| **DMN** | CP | W – 40 | F _(1,40)_ = 0.165, η² = .004 | 0.687 |
| **DMN** | CP | W – 50 | F _(1,40)_ = 3.462, η² = .080 | 0.070 |
| **DMN** | LP | W – 10 | F _(1,40)_ = 0.221, η² = .005 | 0.641 |
| **DMN** | LP | W – 20 | F _(1,40)_ = 0.101, η² = .003 | 0.753 |
| **DMN** | LP | W – 30 | F _(1,40)_ = 0.115, η² = .003 | 0.737 |
| **DMN** | LP | W – 40 | F _(1,40)_ = 5.150, η² = .0114 | 0.029* |
| **DMN** | LP | W – 50 | F _(1,40)_ = 1.190, η² = .029 | 0.282 |
| **DMN** | SWI | W – 10 | F _(1,40)_ = 1.318, η² = .042 | 0.260 |
| **DMN** | SWI | W – 20 | F _(1,40)_ = 0.078, η² = .002 | 0.781 |
| **DMN** | SWI | W – 30 | F _(1,40)_ = 0.880, η² = .022 | 0.354 |
| **DMN** | SWI | W – 40 | F _(1,40)_ = 0.594, η² = .015 | 0.445 |
| **DMN** | SWI | W – 50 | F _(1,40)_ = 1.054, η² = .026 | 0.311 |

**Supplementary Table 4**. Overview of the mixed ANOVA test statistics for the weighted analysis.
WB: whole brain, DMN: default mode network, B: binary graph, W: weighted graph, ^*^: p < .05.

| **Network** | **Pre/Post** | **Graph - threshold** | **Test statistic** | **P-value** | **MC** |
| --- | --- | --- | --- | --- | --- |
| **WB** | Pre | B – 10 | n.a. | n.a. | n.a. |
| **WB** | Pre | B – 20 | n.a. | n.a. | n.a. |
| **WB** | Pre | B – 30 | χ²(1) = 0 | p = 1 | MC = 1 |
| **WB** | Pre | B – 40 | χ²(1) = .359 | p = 1 | MC = 1 |
| **WB** | Pre | B – 50 | χ²(1) = 1.024 | p = 1 | MC = 1 |
| **DMN** | Pre | B – 10 | n.a. | n.a. | n.a. |
| **DMN** | Pre | B – 20 | n.a. | n.a. | n.a. |
| **DMN** | Pre | B – 30 | n.a. | n.a. | n.a. |
| **DMN** | Pre | B – 40 | χ²(1) = 1.024 | p = 1 | MC = 1 |
| **DMN** | Pre | B – 50 | χ²(1) = 2.1 | p = .488 | MC = .492 |
| **WB** | Post | B – 10 | n.a. | n.a. | n.a. |
| **WB** | Post | B – 20 | χ²(1) = 2.1 | p = .488 | MC = .492 |
| **WB** | Post | B – 30 | χ²(1) = 1.024 | p = 1 | MC = 1 |
| **WB** | Post | B – 40 | χ²(1) = 2.1 | p = .488 | MC = .492 |
| **WB** | Post | B – 50 | χ²(1) = 1.024 | p = 1 | MC = 1 |
| **DMN** | Post | B – 10 | n.a. | n.a. | n.a. |
| **DMN** | Post | B – 20 | n.a. | n.a. | n.a. |
| **DMN** | Post | B – 30 | n.a. | n.a. | n.a. |
| **DMN** | Post | B – 40 | n.a. | n.a. | n.a. |
| **DMN** | Post | B – 50 | n.a. | n.a. | n.a. |

**Supplementary Table 5.** Overview of the chi-squared test statistics regarding the parameters present small-world topology and a sustained concussion in binary networks. WB: whole brain, DMN: default mode network, B: binary graph, W: weighted graph, χ = chi-square test statistic, MC: Monte-Carlo simulation significance, ^*^: *p <* .05.

| **Network** | **Pre/Post** | **Graph - threshold** | **Test statistic** | **P-value** | **MC** |
| --- | --- | --- | --- | --- | --- |
| **WB** | Pre | W – 10 | n.a. | n.a. | n.a. |
| **WB** | Pre | W – 20 | n.a. | n.a. | n.a. |
| **WB** | Pre | W – 30 | χ²(1) = 0 | p = 1 | MC = 1 |
| **WB** | Pre | W – 40 | χ²(1) = 0 | p = 1 | MC = 1 |
| **WB** | Pre | W – 50 | n.a. | n.a. | n.a. |
| **DMN** | Pre | W – 10 | n.a. | n.a. | n.a. |
| **DMN** | Pre | W – 20 | n.a. | n.a. | n.a. |
| **DMN** | Pre | W – 30 | n.a. | n.a. | n.a. |
| **DMN** | Pre | W – 40 | χ²(1) = 1.024 | p = 1 | MC = 1 |
| **DMN** | Pre | W – 50 | n.a. | n.a. | n.a. |
| **WB** | Post | W – 10 | n.a. | n.a. | n.a. |
| **WB** | Post | W – 20 | χ²(1) = 1.024 | p = 1 | MC = 1 |
| **WB** | Post | W – 30 | χ²(1) = 2.1 | p = .488 | MC = .487 |
| **WB** | Post | W – 40 | χ²(1) = 5.676 | p = .048* | MC = .046 |
| **WB** | Post | W – 50 | n.a. | n.a. | n.a. |
| **DMN** | Post | W – 10 | n.a. | n.a. | n.a. |
| **DMN** | Post | W – 20 | n.a. | n.a. | n.a. |
| **DMN** | Post | W – 30 | n.a. | n.a. | n.a. |
| **DMN** | Post | W – 40 | χ²(1) = 1.024 | p = 1 | MC = 1 |
| **DMN** | Post | W – 50 | n.a. | n.a. | n.a. |

**Supplementary Table 6**. Overview of the chi-squared test statistics regarding the parameters present small-world topology and a sustained concussion in weighted networks. WB: whole brain, DMN: default mode network, B: binary graph, W: weighted graph, χ = chi-square test statistic, MC: Monte-Carlo simulation significance, ^*^: *p <* .05.

*** If you should have further requests for data, please direct these to CR. Additionally, upon reasonable request with CR, we can send you the standard operating procedure (SOP) for the resting-state EEG measurements and the standardized exercise test:**
Claus Reinsberger; email: [reinsberger@sportmed.upb.de](mailto:reinsberger@sportmed.upb.de)
